# In Vitro Evaluation of GLP-1R-Associated Activity of a Sustainable Standardized Phospholipid-Formulated Bergamot Extract

**DOI:** 10.3390/biomedicines14051111

**Published:** 2026-05-14

**Authors:** Amjad Khan, Rosa M. Mella, Patricia Villacé, Meritxell Roura-Ferrer, Jorge Gamiz, Alessandro Poli, Loredana Redaelli, Giovanna Maresca, Giovanna Petrangolini

**Affiliations:** 1 Department of Biochemistry, Liaquat University of Medical and Health Sciences, Jamshoro 79060, Pakistan; 2 Innoprot, Parque Tecnológico de Bizkaia Edificio 502, Primera Planta, 48160 Derio, Bizkaia, Spain; rmella@innoprot.com (R.M.M.); pvillace@innoprot.com (P.V.); mroura@innoprot.com (M.R.-F.); jgamiz@innoprot.com (J.G.); 3 Cell Biology, Axxam, Openzone, Via Meucci 3, Bresso, 20091 Milan, Italy; 4 Medical Department, Indena S.p.A, Viale Ortles 12, 20139 Milan, Italy; giovanna.petrangolini@indena.com

**Keywords:** bergamot, phospholipid formulation, GLP1-R agonist, Nomad Biosensors™, nutraceuticals, Vazguard™

## Abstract

**Background:** Metabolic syndrome is characterized by dysregulated glucose metabolism and is a major risk factor for type 2 diabetes mellitus and cardiovascular disease. Although glucose-lowering therapies such as glucagon-like peptide-1 receptor (GLP-1R) agonists are effective, their use may be limited by cost, administration route, side effects and tolerability. Bergamot (*Citrus bergamia* Risso et Poiteau) extract, rich in flavanones, has shown favorable metabolic effects in clinical studies, although its mechanisms of action remain insufficiently defined. This study aimed to investigate the potential glucose-modulating mechanisms of a standardized phospholipid-formulated bergamot extract (BP) (Vazguard™) in vitro. **Methods:** GLP-1R activation was assessed in a U2OS cell line expressing cyclic adenosine monophosphate (cAMP)-sensitive Nomad Biosensors™. Dipeptidyl peptidase-4 (DPP4) activity was evaluated using a cell-free enzymatic assay, while Glucose transporter type 4 (GLUT4)-mediated glucose uptake was assessed in CHO-K1 cells stably expressing human GLUT4 using an adenosine triphosphate (ATP)-based readout. Cytotoxicity was also using lactate dehydrogenase (LDH), MTT, and nuclei count assays. **Results:** BP exhibited a dose-dependent (0.31–5 mg/mL) increase in cAMP biosensor fluorescence, consistent with GLP-1R-associated signaling and a maximal response of approximately 60% relative to the positive control (GLP-1R agonist II). No cytotoxic effects were observed. In contrast, BP showed no inhibitory effect on DPP4 activity and did not alter GLUT4-mediated glucose uptake under the experimental conditions tested. **Conclusions:** These findings provide novel mechanistic evidence that phospholipid-formulated bergamot extract suggests a possible involvement in GLP-1R-associated signaling in vitro, without detectable effects on DPP4 or GLUT4 pathways under the conditions tested. This suggests a mechanism consistent with weak agonist or allosteric modulation of GLP-1R and supports further investigation of bergamot formulated with phospholipids as potential natural adjuncts in metabolic health management.

## 1. Introduction

Metabolic syndrome is a major global health concern linked to increased risk of cardiovascular disease, type 2 diabetes mellitus (T2DM), and associated complications including nephropathy, retinopathy, and neuropathy [1,2]. Pharmacological interventions targeting hyperglycemia, such as glucagon-like peptide-1 receptor (GLP-1R) agonists, offer potent glycemic and cardiometabolic benefits [3]. However, limitations including high cost, injectable administration, and side effects—particularly gastrointestinal symptoms and tolerability issues—have hindered widespread long-term adherence [4,5,6,7,8]. These limitations have, in recent years, contributed to growing scientific interest in plant-derived nutraceuticals with glucose-lowering potential [9,10,11,12,13,14,15]. Emerging mechanistic evidence suggests that certain bioactive phytochemicals may modulate pathways already recognized as clinically relevant in metabolic regulation. In addition, these compounds are being explored as potentially more accessible and generally well-tolerated adjuncts.

Among the pathways implicated in glycemic regulation, GLP-1R signaling has emerged as one of the most clinically relevant therapeutic targets [3]. GLP-1R is a G protein-coupled receptor (GPCR) predominantly expressed on pancreatic β-cells and various extrapancreatic tissues. Its activation enhances glucose-dependent insulin secretion, suppresses glucagon release, slows gastric emptying, promotes satiety, and contributes to improved glucose handling with reduced hypoglycemia risk [5,6,7,8,16]. Current GLP-1R therapies include peptide agonists (e.g., Exenatide, Liraglutide) that mimic endogenous GLP-1 hormone by binding simultaneously to the receptor’s extracellular domain and transmembrane core [17,18], thereby stabilizing its active conformation and triggering Gαs-coupled cyclic adenosine monophosphate (cAMP)/Protein kinase A signaling (PKA) signaling [17,18]. Structurally, peptide-based agonists can be categorized into those with a human GLP-1 backbone (e.g., dulaglutide, albiglutide, liraglutide, semaglutide) and those with an exendin-4 backbone (e.g., exenatide, lixisenatide) [3]. More recently, non-peptidic small-molecule agonists such as danuglipron have also been developed that target allosteric sites within the transmembrane domain to fine-tune receptor activity, or activate the receptor independently, further demonstrating that GLP-1R can be pharmacologically modulated by structurally diverse ligands [19,20].

In addition to direct receptor activation, preservation of endogenous incretin signaling represents another important mechanism for improving glycemic control [21,22]. Dipeptidyl peptidase-4 (DPP4) is a serine protease responsible for the rapid degradation of incretin hormones, including GLP-1, thereby limiting their biological activity. Accordingly, inhibition of DPP4 prolongs endogenous incretin action and enhances glucose-dependent insulin secretion. This mechanism is clinically exploited by oral antidiabetic agents such as sitagliptin, saxagliptin, linagliptin, and vildagliptin, which are widely used as glucose-lowering therapies [21,22]. Taken together, GLP-1R agonism and DPP4 inhibition illustrate that the incretin axis can be therapeutically targeted at multiple levels, including both receptor activation and preservation of endogenous ligand availability.

Beyond incretin biology, peripheral glucose handling also plays a central role in glycemic regulation [23,24]. Glucose transporter type 4 (GLUT4) is the principal insulin-responsive glucose transporter in skeletal muscle and adipose tissue, where it facilitates cellular glucose uptake and contributes substantially to postprandial glucose disposal [23,24]. Impaired GLUT4 expression, trafficking, or activity is closely associated with insulin resistance and dysglycemia, making this pathway biologically relevant when exploring the glucose-modulating potential of bioactive compounds. Although GLUT4 is not directly targeted by current antidiabetic drugs in the same way as GLP-1R or DPP4, enhancement of peripheral glucose uptake remains a key downstream determinant of metabolic control and therefore represents another plausible mechanism through which nutraceuticals may exert antihyperglycemic effects. Together, these pathways represent complementary mechanisms involved in the regulation of glucose homeostasis.

Botanical extracts rich in structurally diverse flavonoids have gained increasing interest in recent years as safe and well-tolerated adjuncts, with emerging mechanistic evidence demonstrating their capacity to modulate signaling pathways implicated in glucose metabolism [9,10,11,12,13,14,15]. Natural flavonoids such as quercetin, apigenin, luteolin, and myricetin have been reported to modulate pathways implicated in glucose metabolism, including incretin-related signaling and cellular glucose handling [25,26]. Among flavonoid-rich botanicals of metabolic interest, bergamot (*Citrus bergamia* Risso et Poiteau) extracts, a citrus fruit native to the Calabria region of southern Italy, has emerged as a candidate of increasing interest. It is rich in flavanones such as naringin, neoeriocitrin, neohesperidin, melitidin, and brutieridin [27], and has demonstrated hypolipidemic and glycemic-regulating effects in recent clinical trial studies [11,14,19,20,28,29,30,31,32,33]. These flavonoids, which constitute >95% of the bergamot extract, are considered the primary bioactive components [12]. Accordingly, the health effects of bergamot extracts are believed to arise from the synergistic and/or additive actions of these flavonoid constituents [12]. Bergamot flavonoids have been implicated in modulating metabolic pathways through various mechanisms, including AMP-activated protein kinase (AMPK) activation, cAMP phosphodiesterases (PDEs) inhibition, and 3-hydroxy-3-methylglutaryl coenzyme A (HMG-CoA) reductase mimicry [9,10,11,12,13,14,15]. Despite these encouraging clinical and preclinical observations, the molecular mechanisms underlying the potential glucose-modulating effects of bergamot remain insufficiently defined.

In the present study, we investigated the potential glucose-modulating mechanisms of a sustainable and standardized, phospholipids-formulated bergamot extract (Bergamot Phospholipids or simply BP, Vazguard™), rich in flavonoids such as naringin, neoeriocitrin, and neohesperidin, and previously characterized for its phytochemical profile [27]. Specifically, we assessed whether BP influences GLP-1R signaling, DPP4 activity, and GLUT4-mediated glucose uptake. Cytotoxicity was also evaluated to ensure that any observed responses were not confounded by impaired cell viability or membrane integrity. 

## 2. Materials and Methods

### 2.1. Bergamot Extract

The study utilized a standardized bergamot fruit juice extract (herein as BP, Vazguard™, Indena S.p.A., Milan, Italy), formulated with sunflower phospholipids (Phytosome™, Indena S.p.A., Milan, Italy) to improve polyphenol solubility and optimize absorption [31,32,33,34,35]. The extract was obtained from bergamot juice following fruit separation from its peel and squeezing, and subsequently concentrated and purified using an eco-sustainable water-based extraction process, which avoids the use of organic solvents and is designed to reduce environmental impact. BP has been standardized to contain 14–18% total bergamot flavanones, as determined by high-performance liquid chromatography (HPLC) according to the manufacturer’s validated quality control procedures. The phytochemical profile of bergamot extracts has been previously characterized using advanced analytical techniques, including UPLC-DAD-MS and LC-NMR [27], including major flavanones such as naringin, neoeriocitrin, and neohesperidin.

### 2.2. GLP-1R Activation Assay

The GLP-1R activation screening assay was conducted using the cAMP Nomad-GLP-1R U2OS cell line (Innoprot SL, Derio, Spain), incorporating Nomad Biosensors™ (Innoprot SL, Derio, Spain) [36], a family of genetically encoded fluorescent sensors specifically designed to monitor G protein-coupled receptor (GPCR) signaling. These sensors detect intracellular dynamics of second messengers, such as cAMP, upon GPCR activation. Prior to receptor activation, Nomad Biosensors™ are localized at the plasma membrane, and ligand binding induces a conformational change that enhances fluorescence intensity and triggers intracellular redistribution, reflecting GPCR signaling activity.

Cells were seeded in black, flat-bottom 96-well plates (Becton Dickinson, Franklin Lakes, NJ, USA) at a density of 20,000 cells per well, based on optimized assay conditions for the cAMP Nomad-GLP-1R U2OS cell line to ensure adequate biosensor signal and reproducible fluorescence measurement, and cultured in DMEM-F12 (Sigma-Aldrich, Louis, MO, USA) supplemented with 10% fetal bovine serum (FBS, Sigma-Aldrich, Louis, MO, USA) at 37 °C in a humidified 5% CO_2_ atmosphere for 24 h. BP and phospholipids as vehicle control were solubilized in dimethyl sulfoxide (DMSO) to prepare a stock solution of 500 mg/mL, which was subsequently diluted in assay medium to achieve the final working concentrations. Test solutions, including BP, as well as the GLP-1 receptor agonist II (positive control), were prepared in OptiMEM medium (Thermo-Fisher Scientific, Waltham, MA, USA) and incubated with the cells overnight in triplicates. The GLP-1 receptor agonist II (1 µM) (Sigma-Aldrich, St. Louis, MO, USA) was used as a positive control for assay validation. After incubation with the sample solution, the medium was replaced with 100 µL of phosphate-buffered saline (PBS) to prepare for fluorescence intensity acquisition. Intracellular cAMP levels, proportional to GLP-1R activation, were quantified using the cAMP Nomad Biosensors™ (Innoprot SL), which measures fluorescence intensity changes associated with cAMP signaling. Nuclei were counterstained with Hoechst dye (0.5 µg/mL, Thermo-Fisher Scientific) for 30 min. Fluorescence imaging was performed using the Thermo Fisher Cell Insight High-Content Bioimager, with excitation and emission filters set at 549/15 nm and 640/30 nm, respectively, for the cAMP Nomad Biosensor. Hoechst fluorescence was captured using filters at 386/23 nm and 438/47 nm for excitation and emission, respectively. Fluorescence intensity values were analyzed using Cellomics Scan Version 6.6.4 Software, (Thermo-Fisher Scientific, Waltham, MA, USA) and dose–response curves were generated to calculate EC_50_ values, providing a quantitative measure of GLP-1R activation. 

### 2.3. Cytotoxicity Assays

The cytotoxicity of BP on cAMP Nomad-GLP-1R cell line was assessed using complementary assays evaluating plasma membrane integrity, mitochondrial activity, and total cell count.

#### 2.3.1. Lactate Dehydrogenase (LDH) Assay

Plasma membrane integrity was assessed using the lactate dehydrogenase (LDH) assay (Roche, Basel, Switzerland) according to the manufacturer’s instructions. LDH release, an indicator of membrane damage, was measured in the culture supernatant. Absorbance was recorded at 490 nm using a microplate reader, with higher LDH levels indicating compromised cell membranes.

#### 2.3.2. MTT Assay

Cell viability was further assessed by evaluating mitochondrial activity using the 3-(4,5-dimethylthiazol-2-yl)-2,5-diphenyltetrazolium bromide (MTT) assay (Sigma-Aldrich, St. Louis, MO, USA). Following treatment with the test solution, cells were incubated with MTT solution (0.5 mg/mL) for 3 h at 37 °C. Formazan crystals formed by viable cells were solubilized in dimethyl sulfoxide (DMSO, Sigma-Aldrich St. Louis, MO, USA), and absorbance was measured at 570 nm using the Biotek Synergie II microplate reader (Agilent Technologies, Santa Clara, CA, USA), with background absorbance subtracted for correction. A decrease in absorbance compared to untreated controls indicated reduced metabolic activity and cell viability.

#### 2.3.3. Cell Count Assay

Total cell number was estimated by quantifying Hoechst-stained nuclei. Nuclei were stained with 0.5 µg/mL Hoechst dye for 30 min, and nuclei counting was performed using the Cell Insight High-Content Bioimager CX7 (Thermo-Fisher Scientific, Waltham, MA, USA). Excitation and emission filters were set at 386/23 nm and 438/47 nm, respectively, to visualize and quantify stained nuclei.

This complementary approach ensured that the GLP-1R activation results obtained with BP were not confounded by cytotoxic effects, thereby supporting reliable interpretation of the assay outcomes.

### 2.4. DPP4 Inhibition Assay

To evaluate the potential inhibitory effects of BP on DPP4 activity, a commercial DPP-4 Inhibitor Screening Assay Kit (Abcam, Cambridge, UK, cat. no. ab133081) was used and adapted to white 384-well plates (NUNC, Monza, Italy, cat. no. 460372). The assay employs the fluorogenic substrate Gly-Pro-Aminomethylcoumarin (AMC) to quantify DPP4 activity. Cleavage of the substrate by DPP4 releases free AMC, generating a fluorescent signal measured at an excitation wavelength of 350–360 nm and an emission wavelength of 450–465 nm. Fluorescence was recorded using the PherastarPLUS plate reader, and % activity was calculated relative to the internal controls provided in the kit, including 100 μM sitagliptin at maximal inhibitory concentration as the inhibitor control and assay buffer as neutral control [37]. BP or phospholipids (vehicle control), were both tested in quadruplicates at 8 concentrations up to 100 μg/mL (0.03, 0.1, 0.3, 1, 3, 10, 30 and 100 μg/mL).

### 2.5. GLUT4-Mediated Glucose Uptake Assay

To evaluate whether BP could influence GLUT4-mediated glucose uptake, a CHO-K1 cell model stably expressing human GLUT4 was employed. This assay was designed to assess the potential effects of BP on glucose transport and downstream adenosine triphosphate (ATP) production under conditions favoring GLUT4-dependent cellular glucose uptake.

CHO-K1 cells were maintained in DMEM-F12 with L-glutamine and 25 mM HEPES (EuroClone, Pero, MI, Italy, ECM0095L) supplemented with 10% fetal bovine serum (Merck/Sigma, Darmstadt, Germany, cat. no. F7524), 1% penicillin–streptomycin (EuroClone, Pero, MI, Italy, ECB3001D), Glutamine Stable 100X (BioWhittaker, from EuroClone, Pero, MI, Italy, ECB3004D), 1 mM sodium pyruvate (EuroClone, Pero, MI, Italy, ECM0542D), and 13.5 mM NaHCO_3_ 7.5% (EuroClone, Pero, MI, Italy, ECM0980D).

To generate the GLUT4-expressing model, CHO-K1 cells were transduced with lentiviral particles carrying the pLV[Exp]_Neo_CMV-*hSLC2A4* vector encoding human GLUT4, while cells transduced with pLV[Exp]_EGFP-Puro_EF1A-mCherry served as mock controls. Polybrene was added to the transduction mixture at a final concentration of 8 µg/mL, and cells were centrifuged with the viral suspension at 2000 rpm for 20 min. Cell pellets were then resuspended and seeded into 6-well plates containing 1 mL of fresh medium. After 24 h, the medium containing lentiviral particles was replaced with fresh complete medium, and 48 h later, antibiotic selection was initiated using G-418 (2 mg/mL).

The GLUT4 functional assay was adapted from the ATP-based glucose uptake strategy described by Siebeneicher et al. [38], with modifications appropriate for the present experimental system. Briefly, CHO-K1/hGLUT4 cells were seeded with 20 µL/well of glucose-free DMEM (Life Technologies, Monza, MB, Italy, cat. no. 11966025) supplemented with 2% FBS (Merck/Sigma, Darmstadt, Germany, cat. no. F7524), rotenone (10 µM) to inhibit endogenous ATP production via oxidative phosphorylation, and BAY-876 (100 nM) to inhibit the constitutively expressed GLUT1 transporter. This pre-incubation step (30 min at 37 °C) was used to restrict glucose influx and ATP production predominantly to GLUT4-dependent activity.

BP or phospholipids (vehicle control), both resuspended in DMSO, were then added at 10 µL/well to achieve a final concentration of 100 µg/mL (prepared as 3×, 300 µg/mL, with a final DMSO concentration of 0.5%) and incubated for a further 30 min at 37 °C. As an additional negative control, medium containing 0.5% DMSO alone was used.

Glucose stimulation was then initiated by adding 10 µL/well of D-(+)-glucose in dose response up to 100 mM (prepared as 4×, 400 mM, 8-point 1:2 serial dilution). A fixed 100 mM D-(+)-glucose condition was used as the stimulator control (maximum signal), whereas glucose-free DMEM containing 2% FBS served as the neutral control (minimum signal). Cells were incubated for 1 h at 37 °C.

Plates were subsequently equilibrated to room temperature, and 20 µL/well of CellTiter-Glo 2.0 (Promega, Milan, Italy) was added to quantify intracellular ATP production. Luminescence, proportional to cellular ATP levels, was recorded using either the PherastarPLUS or FDSS7000 Hamamatsu plate readers. Endpoint data were analyzed using Genedata (Version 2026.0.1-Standard) and expressed as % activity or raw luminescence values.

## 3. Results

### 3.1. GLP-1R-Asscociated Signaling

**Figure 1:**
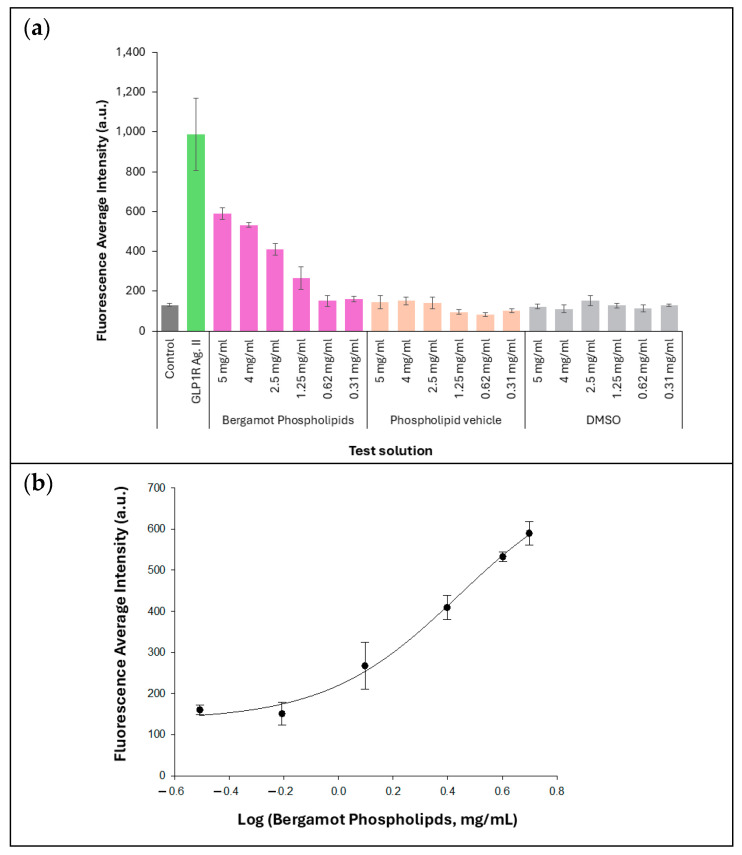
U2OS cAMPNomad-GLP-1R cell-based activation assay. (**a**) Cells were treated with test item solution at increasing concentrations. GLP-1 receptor agonist II (1 µM) was included as a positive control to validate assay performance. Results are presented as mean cAMP Nomad biosensor fluorescence intensity per cell. Each data point represents the mean ± standard deviation (SD) of three replicate wells. The control corresponds to OptiMEM-treated cells. (**b**) Dose–response curve of BP in the GLP-1R activation assay. Values were calculated using logarithmic regression analysis and are presented as mean ± SD of three replicate wells.

BP exhibited a dose-dependent increase in cAMP Nomad biosensor fluorescence intensity across the tested concentration range (0.31–5 mg/mL), reaching an approximately 5-fold enhancement at the highest concentration tested (Figure 1a), consistent with modulation of GLP-1R-associated signaling. The maximal response achieved by BP corresponded to approximately 60% of the effect elicited by the positive control under the same experimental conditions. The half-maximal effective concentration (EC_50_) was calculated as 2.51 ± 0.35 mg/mL, based on the mean of three independent experiments (Figure 1b). At the concentrations tested, neither the phospholipid vehicle control nor DMSO induced any significant increase in fluorescence (Figure 1a), supporting that the observed response was attributable to BP. The GLP-1R Agonist II used as a positive control produced a robust increase in biosensor fluorescence (Figure 1a), supporting the validity of the experimental system.

### 3.2. Cytotoxicity Assessment

The cytotoxicity of BP was evaluated in U2OS cAMPNomad-GLP-1R cell line using three complementary assays across the tested concentration range (0.31–5 mg/mL). No evidence of cytotoxicity was observed in any of the assays performed.

**Figure 2:**
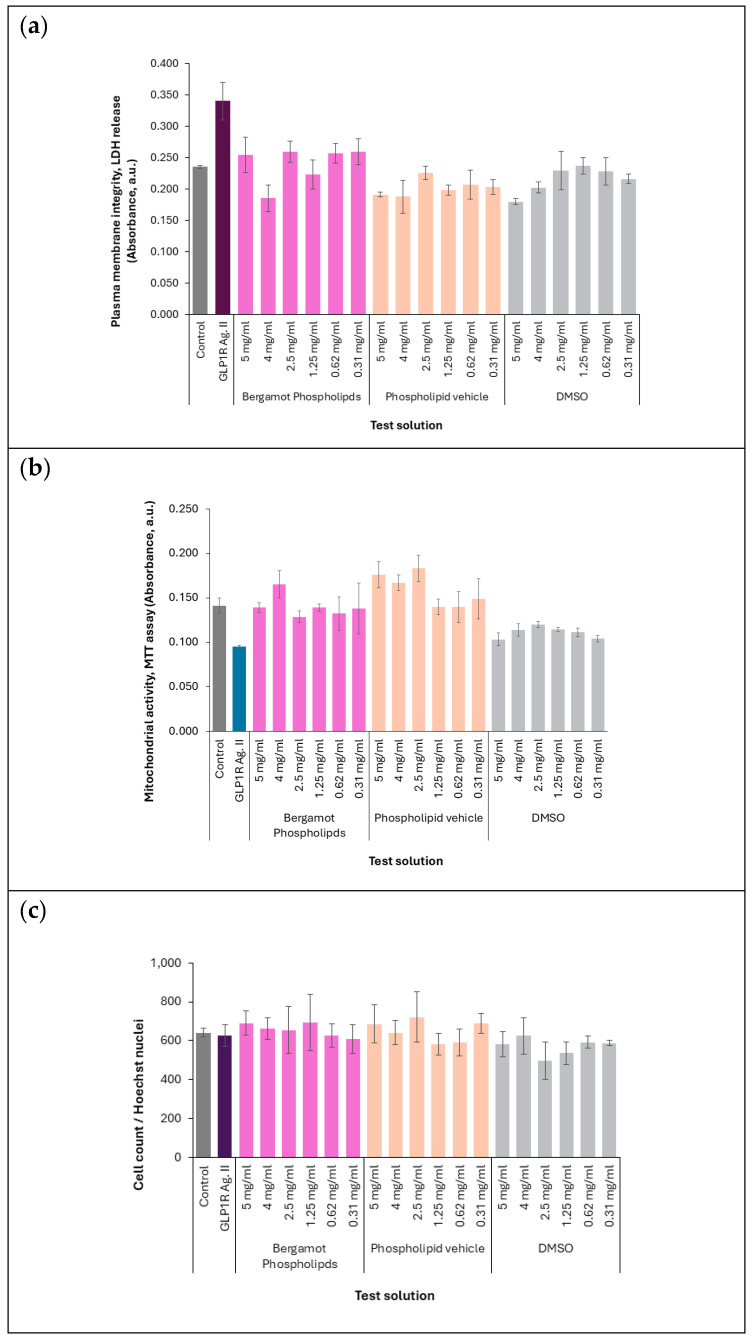
Cytotoxicity evaluation of BP in U2OS cAMPNomad-GLP-1R cells. (**a**) Plasma membrane integrity was assessed using the LDH release assay. (**b**) Mitochondrial activity was evaluated using the MTT assay. (**c**) Total cell number was estimated by nuclei counting. Untreated control cells were included as a reference condition for assessment of cell viability; GLP-1 receptor agonist II-treated cells were also evaluated and showed no cytotoxic effects under the assay conditions. Data are presented as mean ± standard deviation (SD) of three independent experiments.

The LDH release assay (Figure 2a) showed no significant increase in LDH levels at any tested concentration, indicating that BP did not affect plasma membrane integrity under the experimental conditions.

The MTT assay (Figure 2b) further supported these findings by demonstrating comparable mitochondrial activity between BP-treated cells and the untreated control across all tested concentrations. This indicates no significant cytotoxic effects, indicating an acceptable in vitro tolerability profile of BP.

Lastly, the cell count assay (Figure 2c) demonstrated that BP maintained consistent cell numbers relative to the untreated control across all tested concentrations. This further confirms the absence of cytotoxic effects on cell proliferation or survival.

Together, these results provide evidence of an acceptable in vitro tolerability profile of BP, with no adverse effects observed on membrane integrity, mitochondrial activity, or cell number under the tested conditions.

### 3.3. Effect on DPP4 Activity

**Figure 3:**
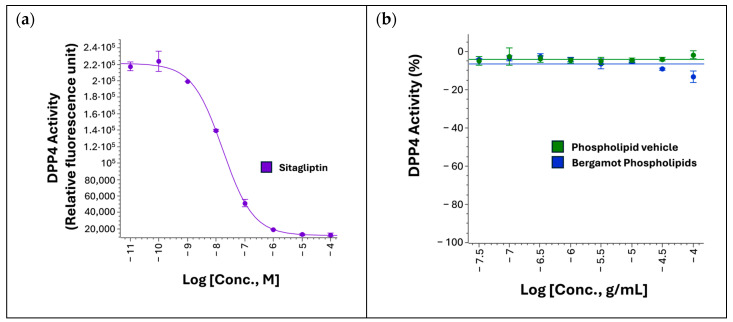
DPP4 inhibitions assay (**a**) Dose–response curve of sitagliptin used to validate assay performance. Data are presented as DPP4 activity measured in relative fluorescence units (RFUs). (**b**) Dose–response evaluation of BP showing no inhibitory effect on DPP4 activity. Sitagliptin was used as a positive control to validate DPP4 inhibition assay performance. Phospholipid vehicle was used as vehicle control. Data are presented as percentage activity normalized to maximal and minimal sitagliptin responses. Dose–response curves were generated using Genedata software (Version 2026.0.1-Standard) and are representative of two independent experiments.

DPP4 functional inhibition was assessed using a cell-free biochemical assay to evaluate the potential inhibitory effects of BP on enzymatic activity. As shown in Figure 3, assay performance was validated using sitagliptin in dose–response (DR) format, which completely abolished DPP4 activity with an IC_50_ of 15 nM (Figure 3a), in line with published and manufacturer-reported values. BP was then evaluated in DR format in doses up to 100 µg/mL (1:3.16 dilution steps) but showed no inhibitory effect on DPP4 activity at any of the concentrations tested (Figure 3b).

### 3.4. Effect on GLUT4-Mediated Glucose Uptake

**Figure 4:**
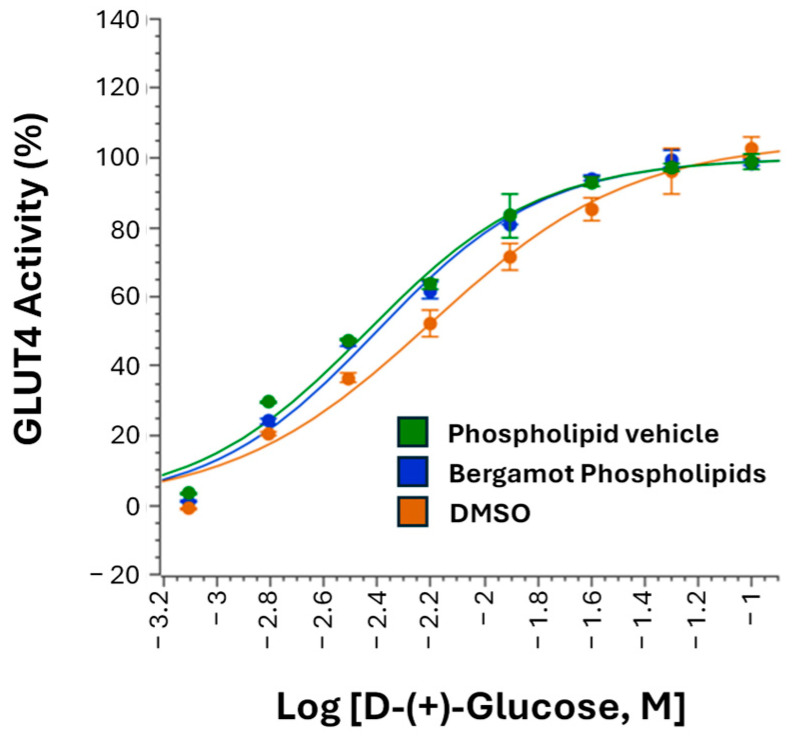
GLUT4-mediated glucose uptake assay. Dose–response curves of D-(+)-glucose in CHO-K1/hGLUT4 cells pre-treated with BP, Phospholipid vehicle, or DMSO control. D-(+)-glucose stimulation was used to drive GLUT4-dependent uptake and served as a functional positive control for assay validation. Data are presented as percentage activity, normalized to maximal and minimal relative luminescence unit (RLU) values recorded in DMSO-treated cells. Curves were generated using Genedata software (Version 2026.0.1-Standard).

A GLUT4 cell-based functional assay was performed to evaluate the potential effects of BP (100 µg/mL) on GLUT4-mediated glucose uptake. As shown in Figure 4, increasing concentrations of D-(+)-glucose (up to 100 mM), used as the functional substrate in the assay, produced the expected dose-dependent increase in ATP signal in CHO-K1/hGLUT4 cells. However, pre-treatment with BP (100 µg/mL) did not alter the glucose dose–response curve compared with DMSO (0.5%) or phospholipid vehicle control, indicating that BP had no detectable effect on GLUT4-mediated glucose uptake under the experimental conditions tested.

## 4. Discussion

The present study provides novel mechanistic evidence that a sustainable and standardized bergamot extract formulated with phospholipids (BP) can activate GLP-1R-associated signaling in vitro within the pathways investigated, while showing no detectable activity on DPP4 enzymatic function or GLUT4-mediated glucose uptake under the experimental conditions tested. Importantly, these effects were observed in the absence of cytotoxicity, supporting the validity of the observed signaling response under the experimental conditions. Taken together, these findings suggest that BP does not exert broad, nonspecific glucose-regulatory activity, but rather may modulate a more preferential incretin-related pathway within the context of the pathways investigated. Furthermore, recent clinical and translational studies continue to highlight both the therapeutic benefits and limitations of GLP-1R-targeted approaches, including challenges related to administration route, tolerability, and variable efficacy across indications [4,5,6,7,8]. These findings are consistent with emerging evidence that plant-derived polyphenols and flavonoids can modulate GLP-1R-related signaling pathways, although challenges related to phytochemical variability, bioavailability, and standardization remain [39].

The most notable finding was the dose-dependent increase in cAMP Nomad Biosensor™ fluorescence observed in the U2OS cAMP Nomad-GLP-1R model. The magnitude of this response, with an approximately 5-fold increase at the highest tested concentration and a maximal effect reaching ~60% of the positive control (GLP-1R agonist II), indicates that BP is capable of activating GLP-1R-associated signaling, albeit with lower efficacy than a pharmacological agonist. This pattern is mechanistically relevant, as it may reflect weak agonism, partial agonism, or allosteric modulation rather than full orthosteric receptor activation. Although the present assay was not designed to resolve receptor binding mode or signaling bias, the observed response profile suggests that bergamot-derived phytochemicals may engage GLP-1R signaling in a measurable manner under the experimental conditions.

This observation is particularly relevant because, despite the growing literature supporting metabolic benefits of bergamot in preclinical and human studies, the precise molecular basis for its potential glucose-modulating effects has remained poorly defined. Previous work has largely focused on mechanisms such as AMPK activation, cAMP phosphodiesterase inhibition, and HMG-CoA reductase mimicry, which have been proposed to contribute to the hypolipidemic and hypoglycemic effects of bergamot polyphenols [31,32,33,34,35]. However, to our knowledge, direct GLP-1R-associated activation by a bergamot extract has not been clearly established. In this context, the present findings extend the mechanistic landscape of bergamot beyond previously proposed metabolic targets and identify incretin-related signaling as a plausible additional pathway of action.

The absence of DPP4 inhibition is equally important for interpreting the GLP-1R-related effect. Because DPP4 inhibition is a clinically validated mechanism for prolonging endogenous GLP-1 activity, it was essential to determine whether the observed signal could simply reflect an indirect incretin-preserving effect. However, BP showed no inhibitory effect on DPP4 activity across the tested concentration range, despite robust assay responsiveness with sitagliptin. This finding narrows the mechanistic interpretation and suggests that the GLP-1R-associated activity observed here is unlikely to be secondary to preservation of endogenous incretin hormones. Instead, the data are more consistent with a receptor-proximal or signaling-level interaction.

Similarly, BP did not alter GLUT4-mediated glucose uptake in the CHO-K1/hGLUT4 assay. GLUT4-mediated glucose uptake represents a major component of peripheral glucose disposal, and many plant-derived bioactives with antihyperglycemic claims are often assumed to act through insulin-sensitizing or glucose transporter-related mechanisms. In the present model, however, BP did not modify the glucose dose–response relationship, indicating that it does not exert an acute stimulatory effect on GLUT4-dependent glucose uptake under these conditions. Together with the lack of DPP4 inhibition, this strengthens the specificity of the overall mechanistic profile and supports the interpretation that BP is not acting as a generalized enhancer of glucose metabolism, but rather as a more preferential incretin-related pathway within the context of the pathways investigated.

The absence of cytotoxicity across all complementary assays further strengthens confidence in the biological relevance of the GLP-1R result. BP did not adversely affect plasma membrane integrity, mitochondrial metabolic activity, or total cell number across the tested concentration range. This indicates that the increase in cAMP biosensor fluorescence was unlikely to result from nonspecific cellular stress, membrane perturbation, or loss of viability. The cytotoxicity data are therefore critical, as they support the conclusion that the observed signaling response reflects a true pharmacodynamic effect rather than an assay artifact.

From a phytochemical and mechanistic perspective, the observed GLP-1R-associated activity is biologically plausible [9,10,11,12,13,14,15,25,26]. Previous studies have shown that natural flavonoids such as apigenin, luteolin, isoquercitrin, and myricetin can stimulate GLP-1 secretion in enteroendocrine cells and increase circulating GLP-1 levels in vivo, thereby contributing to improved glucose tolerance [25]. These observations support the broader concept that dietary polyphenols may influence incretin biology through multiple mechanisms, including both enhancement of endogenous GLP-1 availability and direct modulation of receptor-associated signaling [9,10,11,12,13,14,15,25,26]. Further supporting this premise, Wootten et al. demonstrated that quercetin—a dietary flavonol—can act as an allosteric modulator of GLP-1R, selectively enhancing calcium signaling without affecting cAMP responses [26]. This is particularly relevant in the context of the present findings, as bergamot is characterized by a distinctive flavonoid profile rich in compounds such as naringin, neoeriocitrin, neohesperidin, brutieridin, and melitidin, several of which have been implicated in metabolic regulation or structurally linked to bioactivity in related systems [9,10,11,12,13,14,15,25,26]. Accordingly, the present data fit within an emerging mechanistic framework in which structurally diverse polyphenols may modulate incretin-related pathways. Although the active constituent(s) responsible for the observed effect remain to be identified, it is plausible that the activity reflects additive or synergistic interactions among multiple flavonoids within the bergamot matrix rather than the action of a single dominant compound [31,32,33,34,35].

The use of a phospholipid-based delivery system (Phytosome™) may also be relevant to the observed activity. Bergamot formulation with phospholipids was developed to improve the dispersion and to optimize the bioavailability of bergamot polyphenols, which are otherwise limited by relatively poor solubility. Clinical studies using BP have reported favorable effects on lipid parameters, visceral adiposity, and broader metabolic markers [31,32,33,34,35], supporting the translational relevance of this formulation. Notably, however, these studies have not clearly established the molecular pathways responsible for such effects. In this regard, the present findings are important because they provide a specific mechanistic signal—GLP-1R-associated activation—that may help explain at least part of the metabolic activity previously reported for bergamot-containing formulations [9,10,11,12,13,14,15,25,26,31,32,33,34,35].

At the same time, the relatively high EC_50_ observed in the current assay indicates that the potency of BP is modest, as reflected by the relatively high EC_50_ observed in this assay, when compared with classical pharmacological GLP-1R agonists. This is not unexpected for a complex botanical extract and should not be interpreted as undermining the mechanistic importance of the finding. Rather, it suggests that the biological role of BP is more plausibly aligned with adjunctive metabolic support than with direct pharmacological equivalence to GLP-1R-targeted drugs. This interpretation is also consistent with the broader clinical literature on bergamot [9,10,11,12,13,14,15,25,26,31,32,33,34,35], where benefits have generally been observed as modest but favorable improvements in metabolic and cardiometabolic parameters rather than as drug-like glucose-lowering effects.

Several limitations should be acknowledged. This was an in vitro mechanistic study, and the findings cannot be directly extrapolated to in vivo efficacy. In addition, the current work does not establish whether BP interacts directly with GLP-1R or instead modulates receptor-associated signaling indirectly. The pharmacology was also characterized only through a cAMP-linked readout, and future studies should determine whether BP or its constituent flavonoids exhibit orthosteric, allosteric, partial agonist, or biased signaling behavior across additional GLP-1R pathways, including potential pathway preference involving calcium signaling, β-arrestin recruitment, or other downstream effectors, which represents an important direction for future mechanistic investigation. Furthermore, the active constituent(s) responsible for the observed effect remain undefined. Finally, downstream functional outcomes relevant to incretin biology—including insulin secretion, calcium mobilization, β-arrestin recruitment, and endogenous GLP-1 release—were not assessed and should be explored in future work.

## 5. Conclusions

In conclusion, the present study suggests a possible involvement of GLP-1R-associated signaling within the context of the pathways investigated as a mechanism through which a standardized phospholipid-based bergamot extract may contribute to glucose-modulating effects. The lack of activity on DPP4 and GLUT4, together with the absence of cytotoxicity, is consistent with a more focused, rather than broadly nonspecific, pattern of activity under the experimental conditions. These findings extend current understanding of bergamot’s metabolic bioactivity and provide a rationale for further investigation of bergamot-derived flavonoid formulations in metabolic health.

## Data Availability

The original contributions presented in this study are included in this article; further inquiries can be directed to the corresponding authors.
